# The collaboration of RTs and SLPs in German centers for weaning from mechanical ventilation in neurological and neurosurgical early rehabilitation – results from an online survey

**DOI:** 10.3389/fresc.2026.1766667

**Published:** 2026-06-11

**Authors:** Katrin Eibl, Martin Groß, Martina Hielscher-Fastabend

**Affiliations:** 1 Faculty of Linguistics and Literary Studies, Clinical Linguistics, University of Bielefeld, Bielefeld, Germany; 2 Hospital Barmherzige Brueder Regensburg, Regensburg, Germany; 3 MEDIAN Clinic Bad Tennstedt, Bad Tennstedt, Germany

**Keywords:** early neurological and neurosurgical rehabilitation, mechanical ventilation, multidisciplinary health team, neurorehabilitation, respiratory therapy, speech and language pathology, weaning, collaboration

## Abstract

**Background:**

Weaning in early neurological-neurosurgical rehabilitation (ENNR) is an interdisciplinary process performed by physician-led multidisciplinary teams including speech language pathologists (SLP) and in an increasing number of institutions also respiratory therapists (RT).

**Objective:**

This study analyzes the role, the competencies, the collaboration and their mutual perception of SLPs and RTs involved in ENNR Weaning.

**Methods:**

Two profession-specific web-based surveys (43 items each) were developed to assess work experience, interventions in tracheostomy, communication, secretion and dysphagia management and perceived therapists’ role within the multidisciplinary team. Surveys were distributed to ENNR units and center.

**Results:**

Twenty-seven SLPs and sixteen RTs completed the surveys. SLPs reported more ENNR-specific experience, whereas RTs had more overall clinical experience. Both professions rated their own recommendations—regarding tracheostomy tube selection, timing of cuff deflation, choice of communication methods, and secretion-management strategies—as more important than those of the other group. Both agreed that dysphagia assessment is primarily an SLP responsibility. However, they differed on whether all patients should undergo instrumental examinations such as FEES or tracheoscopy, despite broad agreement on the objectives of these procedures. Both professions regularly trained other team members in tracheostomy management, dysphagia, weaning, and FEES, underscoring their educational roles within the team.

**Conclusion:**

As RTs become more involved in ENNR weaning, enhanced interprofessional education is necessary, particularly regarding communication-focused interventions. Clear job descriptions and SOPs should define shared objectives, procedures, and responsibilities. Training should emphasize above-cuff vocalization, in-line speaking valves (e.g., Passy Muir Valve), and appropriate timing of FEES and tracheoscopy. A targeted educational program for both professions—and for the broader weaning team—may support more efficient weaning, improved communication and swallowing, and better overall patient outcomes.

## 1 Introduction

Centers providing weaning from mechanical ventilation combined with early neurological and neurosurgical rehabilitation (ENNR) have been emerging in Germany during the last two decades. A survey conducted by the German Society for Neurorehabilitation (DGNR) in 2020 found 68 institutions (acute care hospitals and rehabilitation clinics) that could provide around 1,100 weaning beds all over Germany (1). The weaning commission of the DGNR developed a certification for these centers to establish guidelines, framework conditions and high-quality treatment in neurological weaning in 2021 (2).

The centers are generally characterized by having acute care facilities and additionally a multidisciplinary team. This team is key to provide the best possible outcome for critically ill patients, and the benefits are well documented (3–6). Patients with severe neurological or neurosurgical illness are therefore eligible to 300 min of therapy per day, including therapeutic care, physiotherapy, occupational therapy, music therapy, speech and language therapy and - if available - respiratory therapy. Enabling mechanically ventilated patients to speak reduces frustration, fosters motivation, and promotes physiological breathing, thereby supporting the weaning process (7–9). Restoring swallowing function is also a valuable aspect of weaning.

The respiratory therapist (RT) is responsible for the identification, diagnostic, treatment, information, and consultation and care of patients with respiratory diseases according to the German Respiratory Society (DGP) (10, 11). RTs collaborate with physicians, nurses and other allied health professionals. Tasks of the RT in neurological weaning units include diagnostics, managing of secretions and cough, weaning, choosing the tracheal tube type, as well as educating patients and their families (11, 12). In centers providing weaning from mechanical ventilation combined with neurological and neurosurgical rehabilitation the speech and language pathologists (SLP) are responsible for speech, language, voice, and swallowing problems in patients who are on the vent and tracheotomized. Choosing the tracheal tube and managing secretions and cough is also a task of the SLP. In this area, their objectives overlap with those of the RT.

According to their professional education, the SLP is primarily responsible for the functions of the upper whereas the RT is primarily responsible for the functions of the lower airway. In other words, their competencies cross at the larynx which shares both swallowing and respiratory functions. The difference between both groups concerns their training. SLPs have three to four years of education and do not need a further training to work in the ENNR. RTs are a nurse, physiotherapist, occupational therapist or speech language pathologist by profession and then complete a two-year further education. After that they can work as an RT in many different settings.

This survey was conducted to provide insight into the interface and collaboration of RT and SLP in weaning in early neurological-neurosurgical rehabilitation (ENNR) in Germany. Following the certification of these units by the DGNR, and with more and more RTs joining the multidisciplinary team, it was of interest to find out if tasks and objectives of SLP change since they have been working there longer, and how both professional groups would collaborate with each other since a lot of aspects of weaning like cuff deflation, using speaking valves, performing tube changes, utilizing secretion reducing measures and the use of instrumental assessment via FEES and tracheoscopy fall in the hands of both. The fact that FEES is often performed by phoniatricians is not addressed in the survey, as in German ENNR settings the clinicians skilled in FEES are usually neurologists or therapists like SLPs and RTs. Yet, understanding for each other's competencies and responsibilities might differ between SLPs and RTs since before less than a decade ago, the SLPs did not have a RT at their side. The leading research questions were about the perception of each role of SLP and RT in weaning, considering their different competencies and education. Specifically, the study aimed at answering the following questions:

1. How do SLPs and RTs collaborate within a weaning unit in ENNR settings?
2. How do these professional groups perceive the extent of their influence on decisions related to tracheostomy management, facilitation of patient communication, and secretion management?
3. How are both professions organisationally integrated into the multidisciplinary team?

## 2 Materials and methods

### 2.1 Study design

Two anonymous self-administered web-based exploratory surveys were conducted. This approach allows an effective and standardized way to collect data from participants in this specialized setting. The study aims to detect group differences in their subjective perspective of the shared professional field.

### 2.2 Survey development

The surveys were developed for the platform Unipark and piloted using the expertise of two speech language pathologists, one respiratory therapist and a neurointensivist working in centers providing weaning in early neurological and neurosurgical rehabilitation with experience in trach management, dysphagia and research. The neurointensivist respectively coauthor of this article, Martin Gross, is also lead of the certification commission of the DGNR.

The survey items included closed-ended questions, multiple-choice questions, matrix formats, ordinal rating scales, and free-text fields for comments or “other” responses. The answer options for multiple-selection items related to consults, trainings, and diagnostic objectives were developed based on evidence-based literature, established clinical practice, and input from the piloting group.

Both surveys comprised a total of 43 questions. Demographic data like age and gender was not asked for to keep it anonymous. The first questions concerned work experience and experience with tracheostomized and mechanically ventilated patients. Here, the surveys differed. Since respiratory therapy is an additional qualification rather than a standalone profession, RTs have diverse professional backgrounds. Moreover, RTs work exclusively with ventilated patients, whereas SLPs also treat tracheostomized patients who no longer require mechanical ventilation. There were six questions about participation in ward and morning rounds, and trainings to other health professionals. 15 questions covered methods in weaning from mechanical ventilation that are being employed at the interface of the upper and lower airway. The questions were specific about methods in tracheostomy management (choice and change of tube, cuff deflation), communication (use of above cuff ventilation (ACV), use of speaking valves, use of leak speech), dysphagia assessment, secretion management (cough training, respiratory therapy, use of secretion reducing methods), and the use of flexible endoscopic evaluation of swallowing (FEES) and tracheoscopy.

There were 13 ordinal scales to be answered: A five-point-Likert scale from never (1) to always (5) for questions about who is performing the procedure. And a seven-point-Likert-scale from none (1) to the most (7) for questions concerning their influence on recommendations in trach, communication, secretion management and dysphagia assessment.

The last section of the survey concerned the existence of Standard Operating Procedures (SOPs). According to the requirements of the certification of ENNR weaning units, five SOPs are obligatory (2): Weaning, Tracheostomy management, Dysphagia management, Decannulation, Communication management, which were requested.

The study and survey was approved by the Ethics Board of the University of Bielefeld, Germany (2021-257).

### 2.3 Recruitment and participants

The recruitment of participants took place over 4 months (15 June–30 September 2022). The survey was distributed to all neurological rehabilitation clinics and units all over Germany caring for mechanically ventilated patients via the mailing list of the DGNR (German society for Neurorehabilitation). Additionally, it was distributed via the mailing list of the German Society of academic speech and language therapists (dbs). More recruitment took place via a mailing list of respiratory therapists (DGpW) and snowballing via social media (facebook, LinkedIn). There were no incentives provided to participate in the survey.

The surveys targeted RTs and SLPs employed in departments of early neurological–neurosurgical rehabilitation (ENNR) in either acute-care hospitals or rehabilitation clinics who were involved in the care of mechanically ventilated patients in Germany. Respondents who did not work in ENNR settings, as well as those employed in ENNR departments without a weaning unit, were excluded. A total of 39 SLPs initiated the survey, of whom 27 completed it, yielding a completion rate of 67.5%. Among RTs, 24 participants began the survey and 16 completed it, corresponding to a completion rate of 53.3%. Survey dropouts occurred at various points and did not follow a systematic pattern.

Only responses from those who completed the survey in full were included in the analysis.

### 2.4 Data analysis

The data was exported from Unipark to Microsoft Excel and analyzed using the SPSS 26 software (IBM). Demographics such as work experience, work frequency and trainings were analyzed using descriptive statistics to determine frequencies and percentages. Pearsońs-Chi^2^ test and non-parametric Mann–Whitney-U Tests in a 2 × 2 analysis of variances were used to test the scores of the Likert scales. All tests were two tailed and a *p*-value < .05 was considered significant in single statistical tests, and it was adjusted to *p* < .01 for multiple statistical comparisons.

## 3 Results

A total of 43 questionnaires were completed: 27 by SLPs (completion rate 67.50%) and 16 by RTs (completion rate 53.33%). The median completion time was 13 min for the SLP survey and 18 min for the RT survey. Dropouts in both surveys occurred at varying points and did not follow a systematic pattern.

Work experience for both professional groups is presented in Table 1. Among the 16 responding RTs, 11 were trained as general nurses, one as a geriatric nurse, one as a pediatric nurse, two as SLPs, and one as a physiotherapist prior to qualifying as RTs.

**Table 1:** Work experience.

Work experience in years			
total (*n* = 43)	<5 years	5–10 years	>10 years
as an SLP (*n* = 27)	8 (29.6%)	8 (29.6%)	11 (40.7%)
SLP work experience with MV patients	14 (51.9%)	9 (33.3%)	4 (14.8%)
SLP work experience with trach patients	12 (44.4%)	7 (25.9%)	8 (29.6%)
RT in his/her former profession		1 (6.3%)	10 (93.8%)
as an RT (*n* = 16)	9 (56.3%)	7 (43.8%)	
as an RT in ENNR	9 (56.3%)	6 (37.5%)	1 (6.3%)

SLP, speech language pathologist; RT, respiratory therapist; MV, mechanically ventilated, pat., patients; trach, tracheotomized; ENNR, neurological-neurosurgical early rehabilitation.

With regard to work schedules, weekend shifts are common in most rehabilitation clinics. Among SLPs, more than half (55.6%; *n* = 15) reported working only on weekdays, while one quarter (25.9%; *n* = 7) also worked on Saturdays. In contrast, RTs most frequently reported working less than daily (37.5%; *n* = 6) or only on weekdays (31.3%; *n* = 5). Only a small proportion of respondents reported working every day from Monday through Sunday—25.9% of SLPs (*n* = 7) and 12.5% of RTs (*n* = 2).

### 3.1 Collaboration

Regarding collaboration between RTs and SLPs, one third of the SLPs (33.3%; *n* = 9) reported that there were no RTs available for collaboration at their workplace. Fourteen SLPs (51.9%) indicated regular collaboration with RTs, while four (14.8%) reported collaborating irregularly. Among RTs, eleven (68.8%) stated that they collaborated regularly with SLPs, and five (31.3%) reported irregular collaboration. From these 11 RTs who regularly cooperate with SLPs, seven (63.6%) have more than five years of work experience in the ENNR. Based on these responses, significantly more RTs reported collaborating with SLPs than vice versa (*χ*^2^ = 7.123, df = 2, *p* = .028).

When asked who conducts swallow screenings in patients with tracheostomy, both professional groups unanimously agreed that SLPs should perform these screenings (100%). However, 25% of RTs additionally considered nurses responsible for dysphagia screening, and 43.8% reported that they also performed swallow screenings themselves. In comparison, only 18.5% of SLPs reported that nurses conduct dysphagia screenings, and 11.1% stated that RTs should conduct swallow screenings.

For assessing aspiration risk in tracheostomized patients, 81.5% (*n* = 22) of SLPs reported using the Modified Blue Dye Test (MBDT).

Regarding instrumental assessments—specifically FEES (flexible endoscopic evaluation of swallowing) and tracheoscopy—participants were asked whether FEES should be recommended for every patient. The results indicate that RTs were more likely than SLPs to recommend routine FEES use, with a significant difference between groups (*χ*^2^ = 7.123, df = 2, *p* < .028). Conversely, tracheoscopy was recommended more often by SLPs, although this difference was not statistically significant (*χ*^2^ = 1.63, df = 1, *p* = .202). A multiple-choice question listed the potential objectives for performing FEES and tracheoscopy, respectively (see Table 2).

**Table 2:** Objectives of a FEES or tracheoscopy. Values represent the number of raters.

Objectives of instrumental assessment	SLP (%)	RT (%)
FEES objectives		
assessment of swallowing	100	93.8
before oral intake	88.9	68.8
assessment of readiness for decannulation	85.2	100
inspection larynx/pharynx	85.2	87.5
assessment of secretions	85.2	56.3
before diet changes	63.0	37.5
assessing acv	29.6	6.3
Other: position of trach; before first cuff deflation; during cuff deflation		
Tracheoscopy objectives		
inspection trachea/bronchi	92.6	68.8
inspection position of trach	88.9	75.0
assessment of secretions	74.1	43.8
retrograde assessment of subglottic structures	55.6	56.3
before decannulation	48.1	81.3

### 3.2 Organisation within the multidisciplinary team

Not all tracheotomized or mechanically ventilated patients were routinely assessed by an SLP or RT. According to respondents, SLPs evaluated 81.5% of tracheostomized patients and 70.4% of mechanically ventilated patients, whereas RTs assessed 87.5% of patients in both groups.

Participants selected predefined indications for requesting an SLP or RT consultation. Both professions endorsed these indications, although with different frequencies and priority patterns (Table 3).

**Table 3:** Reasons for consults.

Reason for consults	Consults for SLP	Consults for RT
Reasons for consults	oral intake (100%)	ventilator settings (87.5%)
	communication (96.3%)	choice of trach tube (87.5%)
	FEES (92.6%). choice of trach tube (85.2%) further diagnostics (33.3%)	referrals to and outpatient ventilation (81.3%) cuff deflation (75%)
		respiratory therapy (75%)
		non-invasive ventilation (68.8%)
		outpatient settings (62.5%)
Further reasons given	decannulation (3.7%)	cough assist (6.3%)
	cuff deflation (3.7%)	dysphagia therapy FEES (6.3%)
		weaning protocol (6.3%).

Regular interdisciplinary exchange is essential for coordinated care. All RTs (100%) reported participation in ward rounds, whereas only 59.3% of SLPs did so. This difference was statistically significant (*χ*^2^ = 15.339; df = 2; *p* < 0.001). Participation in morning rounds did not differ significantly between groups (SLPs 81.5%; RTs 75.1%; *χ*^2^ = 5.183; df = 3; *p* = 0.159).

The final part of the survey addressed contributions to staff training. No significant differences emerged regarding whether SLPs and RTs provided educational sessions to other health professionals (*χ*^2^ = 0.717; df = 1; *p* = 0.397). Most respondents offered training regularly (SLPs 85.2%; RTs 93.8%). SLPs most frequently taught dysphagia (85.2%), tracheostomy management (70.4%), oral intake and diet (48.1%), swallow screening (44.4%), speech and language disorders (29.6%), and, less commonly, FEES or mechanical ventilation (14.8%). RTs most often taught mechanical ventilation (93.8%), secretion management (93.8%), respiratory therapy (87.5%), tracheostomy management (81.3%), and occasionally additional topics (12.5%) such as dysphagia management, device instruction, or newcomer orientation.

Respondents also reported on the availability of Standard Operating Procedures (SOPs). In accordance with DGNR certification criteria, five SOPs are required: weaning, tracheostomy management, dysphagia management, decannulation, and communication management. Most participants indicated that these SOPs were implemented in their institutions (Table 4). Additional SOPs named in free-text responses included protocols for FEES, oral intake in tracheostomized patients, inhalation therapy, and difficult airway management.

**Table 4:** Existing SOPs.

Existing SOPs According to	Weaning	Tracheostomy management	Dysphagia-management	Decannulation	Communication management	No SOPs
SLP	85.2%	88.9%	74.1%	66.7%	7.4%	0
RT	68.8%	62.5%	56.3%	56.3%	18.8%	18.8%

### 3.3 Perspective on perceived professional role in both groups

In the subsequent sections of the survey addressing the interface between RTs and SLPs in the weaning context, five-point and seven-point Likert scales were used. A 2 × 2 rating structure was applied, in which each professional group (SLPs and RTs) provided repeated-measures evaluations of both their own profession and the other profession across several tasks. This resulted in four rating perspectives: SLPs rating SLPs, SLPs rating RTs, RTs rating RTs, and RTs rating SLPs.

Since a third of SLPs (*n* = 9) do not cooperate with RTs in their clinical setting, these SLPs were excluded in the following analyses.

Respondents rated the perceived influence of each professional group on decisions related to tracheostomy management, communication, and secretion management on a seven-point scale ranging from 1 to 7, with 7 indicating the greatest level of influence. Additional items assessed the frequency with which each group performed specific tasks, using a five-point scale ranging from “never” (1) to “always” (5). The results are summarized in Table 3.

When recommending a tracheostomy tube, both professional groups rated their own influence on these decisions higher than that of the other group (interaction: F(1,32) = 4.73; *p* = .037).

With regard to changing the tracheostomy tube, both SLPs and RTs reported that they are not always the primary professionals performing this task, noting that nurses and physicians are also involved. Nevertheless, the difference between self- and other-ratings remained significant, indicating that RTs are viewed—by both groups—as being most responsible for tube changes and as performing them more frequently (see Table 5).

**Table 5:** Group differences in perceived influence on decision-making and intervention performance in tracheostomy, communication, and secretion management. Values represent mean ratings (SD). Higher scores indicate greater perceived influence. Interaction effects reflect rater×rated-profession differences from two-way repeated-measures ANOVA.

A. Influence on decision-making for SLP partial group (*n* = 18)/RT (*n* = 16) (7-point Likert scale: 1 = none, 7 = highest influence)					
Decision domain	SLP rating SLP	SLP rating RT	RT rating SLP	RT rating RT	Interaction F; p
Type and size of tube	5.56 (1.69)	5.17 (1.86)	4.50 (2.19)	6.00 (1.16)	F(1,32) = 4.73; *p* = .037
Use of ACV	6.75 (0.46)	4.25 (2.25)	3.56 (2.70)	5.56 (1.51)	F(1,22) = 8.58; *p* = .01
Use of PMV	6.36 (0.93)	4.36 (2.44)	4.80 (2.51)	5.67 (1.68)	F(1,27) = 9.04; *p* = .006
Use of one-way valve (OWV)	6.61 (0.70)	3.44 (2.41)	5.63 (1.70)	5.75 (1.07)	F(1,32) = 16.48; *p* < .001 (& main effect within)
Cuff deflation	6.17 (1.30)	3.67 (2.45)	6.13 (1.26)	5.25 (1.57)	Main effects within and between; no sig. interact.
Secretion-reducing methods	5.22 (1.96)	3.94 (2.67)	4.13 (2.22)	6.06 (1.00)	F(1,32) = 10.67; *p* = .003

B. Influence on performance of interventions for SLP partial group (*n* = 18)/RT (*n* = 16) (5-point Likert scale: 1 = never, 5 = always)					
Intervention	SLP rating SLP	SLP rating RT	RT rating SLP	RT rating RT	Interaction F; p
Tube change	3.11 (1.32)	3.33 (1.61)	2.44 (1.50)	3.81 (1.17)	F(1,32) = 4.46; *p* = .043 (& main effect within)
Cough training	3.28 (1.23)	4.00 (1.37)	2.13 (1.36)	4.56 (0.63)	F(1,32) = 7.28; *p* = .011 (& main effect within)
Secretion management	2.33 (1.19)	3.39 (1.67)	2.13 (1.50)	4.31 (0.79)	(only main effect within)

Test of main effect of within subjects contrasts means the comparison of ratings for SLP vs. ratings for RT over both groups; mean effect between subjects contrasts means the comparison of the two groups for all ratings over both variables.

**Figure 1:**
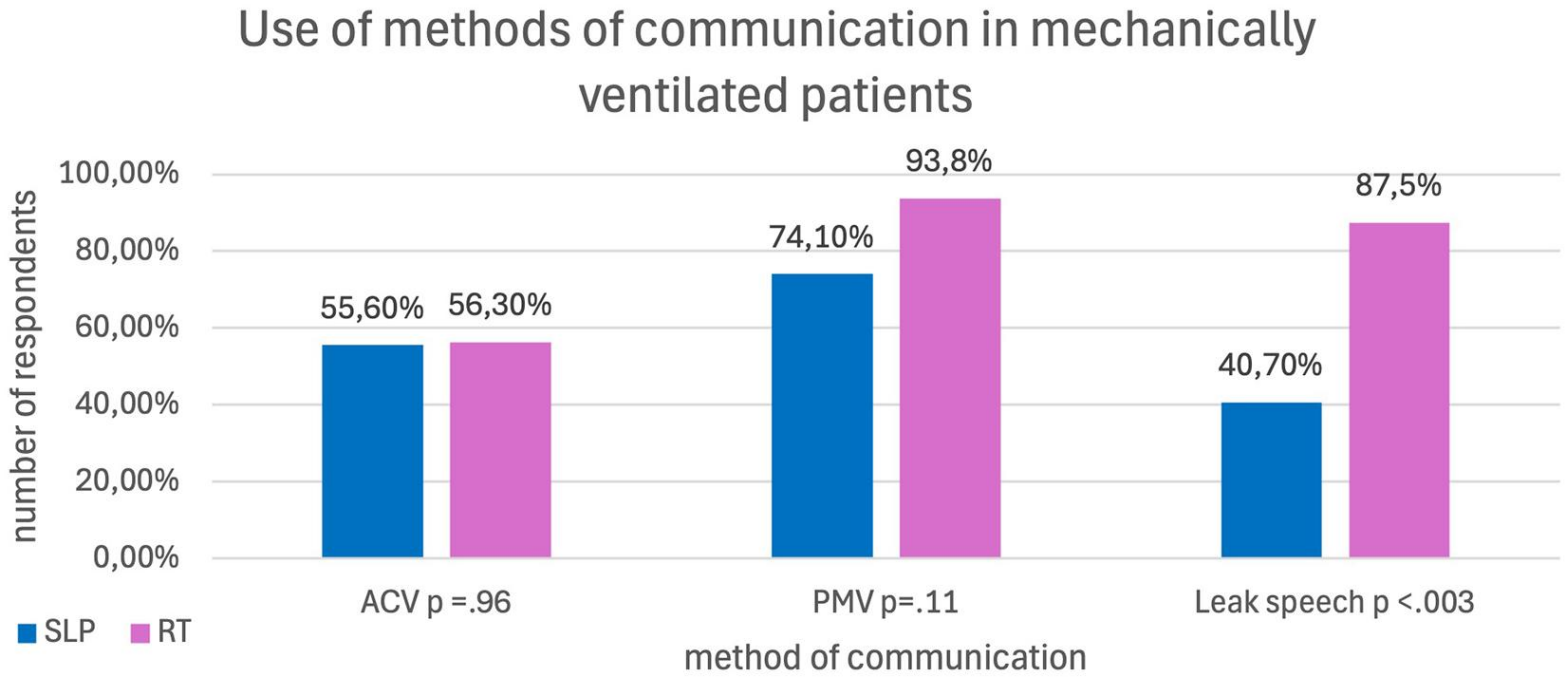
Use of communication methods in mechanically ventilated patients. Values represent the number of respondents using each method. Shown are the use of above-cuff vocalization (ACV), Passy Muir Valve (PMV), and leak speech by speech-language pathologists (SLPs; *n* = 27) and respiratory therapists (RTs; *n* = 16). Group differences were analyzed using chi-square (*χ*^2^) tests.

Several methods are available to enable speech in mechanically ventilated patients, including above-cuff vocalization (ACV), the use of an in-line Passy Muir Valve (PMV), leak speech, and the use of a one-way speaking valve while disconnected from the ventilator. Notably, both groups reported using ACV and in-line PMV only very rarely, with no significant differences between SLPs and RTs. For PMV use, there was a slight trend toward more frequent use among RTs. In contrast, a significant difference emerged for leak speech, with RTs reporting its use more frequently than SLPs (see Figure 1).

With regard to communication methods, the analysis of variance indicates that SLPs are perceived as having greater influence in this domain. Both professional groups agreed that SLPs exert stronger influence across all communication-related decisions. The interaction of ratings revealed a significant difference between the perceived influence of SLPs and RTs in the use of a PMV and the use of a one-way speaking valve. With the use of ACV there was a trend towards a significant difference, and regarding the decision on cuff deflation, both groups seem equal (see Table 5).

Finally, for tasks such as conducting cough training and recommending secretion-reduction strategies, a significant difference emerged in favor of the RTs. Regarding the performance of secretion management both groups consider themselves equally important (see Table 5).

## 4 Discussion

To our knowledge, this is only the second study to systematically examine collaboration between SLPs and RTs in the context of tracheostomy and ventilator weaning. An earlier U.S. survey involving six RTs and six SLPs highlighted several strategies to strengthen collaboration, including interprofessional education, structured communication opportunities, and protocol development (13). In both the previous study and the present one, the complexity and specificity of procedures—many of which are not yet standardized across clinical settings—may have posed challenges for respondents, potentially contributing to survey dropout. This could be explained by careless responding (14).

In our sample, SLPs tended to have more than ten years of ENNR experience, reflecting the historical development of weaning units and the more recent establishment of RT roles within these centers. The DGNR's 2021 certification criteria, which require the presence of an RT, likely contributed to the increasing integration of RTs in ENNR (15).

Both groups have different working schedules. The working schedules of the RTs is far more variable which can make collaboration more difficult if both groups rarely are present at the same time.

### 4.1 Collaboration

A key finding is that significantly more RTs reported collaborating with SLPs than vice versa. It can also be noted, that those RTs who regularly work with SLPs tend to have more than five years of work experience in the ENNR. Therefore, they are used to SLPs being present and working alongside them. One-third of SLPs indicated that they had no RT available in their unit, indicating variability in staffing across institutions. In line with the earlier U.S. study (13), interprofessional interactions remained infrequent in some centers, despite shared patient populations and overlapping responsibilities.

### 4.2 Organisation within the multidisciplinary team

Instrumental assessments such as FEES and tracheoscopy are now considered standard in neurological weaning (16–18). These tools assist in evaluating swallowing, airway protection, and tracheostomy tube positioning, and should ideally be available for all dysphagic tracheostomized patients (19). Limited availability, insufficient staffing, or reliance on clinical assessment alone may explain why these procedures were not routinely used for every patient.

Training and education patterns reflected the scope and expertise of each profession. The universal involvement of RTs in ward rounds likely reflects their structural integration into medical or nursing departments. In contrast, lower SLP participation in ward rounds aligns with historical trends showing that SLPs have not been consistently included in interdisciplinary rounds (20). Their higher involvement in morning rounds led by nursing or therapy staff suggests alternative mechanisms for information exchange (21).

The widespread presence of SOPs reported by respondents indicates a high degree of alignment with DGNR certification requirements. This suggests increasingly standardized care processes and a strong foundation for interprofessional collaboration (22).

### 4.3 Perspective on perceived professional role in both groups

Although both professions work routinely with tracheostomized and mechanically ventilated patients, not all such patients were assessed by an SLP or RT, despite both professions being available on most weekdays. This gap mirrors findings from intensive care units where SLP involvement in tracheostomized patients often occurs late—sometimes 14 days post-tracheostomy (23, 24). Clinical instability or palliative contexts may partly explain these omissions, though earlier involvement would be expected due to the high incidence of dysphagia associated with tracheostomy and mechanical ventilation (16, 25).

Role overlap was evident in tracheostomy management, communication facilitation, secretion management, and tube selection. While swallowing and communication are core components of SLP practice, tube choice and cuff management require shared decision-making involving both SLP and RT expertise. Both professional groups nevertheless rated themselves as having the highest influence across all domains, while rating the other group's influence lower. This perception gap may stem from limited awareness of the other profession's competencies, historical role dominance (particularly among SLPs in units without RTs), or uncertainty regarding the redistribution of responsibilities following the integration of RTs into ENNR teams.

Secretion management is clearly a shared domain. Tracheobronchial secretion retention may result from aspiration, ineffective cough, mucus hypersecretion or viscosity changes, and impaired mucociliary clearance. Addressing these factors requires combined expertise in upper and lower airway function, making interprofessional collaboration essential.

Dysphagia management remains a core SLP responsibility. Nevertheless, nearly half of RTs reported believing that they—and nurses—could perform swallow screenings, whereas only a minority of SLPs supported cross-professional screening. Given that SLP availability is not always immediate, training nursing staff in validated dysphagia screening tools may be beneficial and is supported by existing evidence (16, 25). Incorporating such training, as proposed by Schefold et al. (25), may accelerate dysphagia detection and improve patient flow.

With regard to communication methods, both professions reported low familiarity or use of Above-Cuff Vocalization (ACV), despite the technique's renewed clinical relevance (26–28). ACV allows ventilated patients to phonate without cuff deflation, potentially facilitating communication earlier in the weaning process (28). This underscores the need for joint training initiatives on communication facilitation techniques (29).

### 4.4 Limitations

The low number of respondents might be a limitation, but as the basic population of SLPs and RTs who work in these ENNR weaning units is unknown we cannot provide a response rate (22). The certification of these units will provide numbers, because RTs are obligatory, but these started roughly at the same time of the survey.

For ethical and data protection reasons it was not possible to identify paired samples of SLPs and RTs from the same clinic respectively, which would allow more direct comparisons of the interprofessional view of both professional groups. Furthermore, the survey did not address the fact that in some institutions, other professionals, such as occupational therapists, may be responsible for dysphagia and tracheostomy management. In addition, phoniatricians involved in tracheostomy management and dysphagia diagnostics were not included, as they are very rarely present in ENNR settings in Germany. These professions were not surveyed.

## 5 Conclusion

With more RTs entering the specialized field of collaboration between SLPs and RTs in the context of tracheostomy and ventilator weaning there is a need for creating a common ground on which both professions can use their expertise to the best of their abilities (30). Weaning is a multidisciplinary task with RT and SLP working at a crucial point. Their tasks overlap at the interface of the upper and lower airway. They are both responsible for breathing, swallowing and speaking. Job descriptions that define the objectives at this interface are needed as well as SOPs that specify the procedures, the timing and the responsibilities of both when they attend to patients in weaning. More education is needed especially about the use of above cuff vocalization, the use of in-line speaking valves like the Passy Muir Valve and about the necessity to employ fees and tracheoscopy at the right moment for every patient. To accelerate the weaning process, the recovery and to improve communication and swallowing in every patient a further education program should be developed aiming at exactly these topics for both professions respectively and for all professions involved in weaning.

## Data Availability

The original contributions presented in the study are included in the article/Supplementary Material, further inquiries can be directed to the corresponding author.
