# Supplementary material for: The collaboration of RTs and SLPs in German centers for weaning from mechanical ventilation in neurological and neurosurgical early rehabilitation – results from an online survey
Source: Front Rehabil Sci. 2026 Jun 11;7:1766667. doi: 10.3389/fresc.2026.1766667 (PMC13294173; doi:10.3389/fresc.2026.1766667)
Supplement: Supplementary file 1 [file Datasheet1.pdf]

Questionaire SLP

1 Start page

Dear Participant,
Thank you very much for your interest in the study on the topic:
"The Use of Speech and Language Therapy Interventions for Ventilated Patients in Weaning Centers for Early Neurological and Neurosurgical Rehabilitation (ENNR) in Germany."
If you work as a speech-language pathologist at a facility for Early Neurological-Neurosurgical Rehabilitation (ENNR) with a weaning unit, I would appreciate it if you could complete the following questionnaire in its entirety.
The study has received ethical approval from the University of Bielefeld. Your data will be treated confidentially and used exclusively for research purposes.
The data will be analyzed anonymously, so that no conclusions can be drawn about your identity. Your participation is voluntary, and answering the questions will take approximately 15–20 minutes of your time. If you have any questions or suggestions, you can reach me at the following email address:
katrin.eibl@uni-bielefeld.de

With best regards,
Katrin Eibl

Hereinafter, the term “speech-language pathologists” is used throughout. This refers to all practitioners across all professional groups within speech-language pathology/academic speech and language therapy and logopedics.
The term “respiratory therapists” refers to practitioners of this distinct profession who have completed advanced training in accordance with the DGP or DGPW standards.

2 SLP Survey

How much work experience do you have?

- Less than 1 year
- 1-4 years
- 5-10 years
- More than 10 years

How long have you been working with trach patients?

- Less than 1 year
- since 1-4 years
- since 5-10 years
- since more than 10 years

How long have you been working with mechanically ventilated patients?

- Less than 1 year
- since 1-4 years
- since 5-10 years
- since more than 10 years
- since the begin of the pandemic 2020

Are you working together with respiratory therapists?

- Yes, regularly

- Yes, irregularly
- There are no respiratory therapists, yet

3 SLP Frequency
How often do you work in the ENNR?
- daily (Mon – Sun)
- working days (Mon – Sat)
- working days (Mon - Fri)
- not daily (1—3x /week)
- only on request (when)

4 SLP in ENNR
Are you being consulted for every tracheotomised patient?
- Yes, für every trach patient
- No, only for some trach patients, e.g.
- ______________________________

Are you being consulted for every mechanically ventilated (MV) patient?
- Yes, for every mv patient
- No, only for some mv patients, e.g.
- _______________________________

When are you being consulted?
Multiple choice answers
- Concerning nutrition (diet advancement, NGT, PEG-tube)
- Concerning communication (with/without tracheal cannula)
- Concerning the trach tube (model, size, tube change)
- Concerning/using FEES (execution, assistance, report)
- Concerning further diagnostics (mBS, VFS, radiology, gastroscopy)
- Other:

5 SLP ward rounds
Are you taking part in medical ward rounds?
- Yes, regularly
- Yes, irregularly
- No

Are you taking part in short team meetings in the mornings?
(Short meetings that do not take part at the bedside and last maximum 15-20 min.)
- Yes, regularly
- Yes, irregularly
- No
- There are no such meetings

6 Trainings
Do you conduct trainings?
- Yes
- No

Who do you provide trainings for?
Multiple choice answers

- Medical personnel
- Nursing
- Allied health professionals (Respiratory therapy, Physiotherapy, Occupational therapy, and others)
- There are no trainings offered

What topics are covered in your trainings?
Multiple choice answers

- Dysphagia
- Trach management
- Diet and Nutrition
- Standardised Swallowing assessments
- Speech and language disorders
- Further:

How often do you conduct trainings?

- Regularly, 1x per quarter
- Irregularly, 1 – 3 x year
- Only on request

7 Introduction of concepts and methods
This set of questions focuses on therapeutic methods for ventilated patients, particularly those at the intersection of speech therapy and respiratory therapy.

Although physical therapy, for example, is often involved, it is not included here.

Many decisions regarding specific methods are made by the entire multidisciplinary team. Although decisions must always be made on an individual basis for each patient, standard situations are to be depicted here. To determine which professional group has the greatest influence on the decision regarding a particular intervention, the question "Who recommends…?" is repeatedly asked. You can answer these questions using a rating scale. In doing so, the influence of each professional group is weighted.

8 Management of tracheal cannulas
Under what conditions do you work with trach patients?

- When they are on the ventilator
- Only when they are disconnected from the ventilator
- Only, once they are weaned from the ventilator

9 Matrix management tracheal cannulas
Who recommends the model and size of the tracheal cannula?
Please rate the influence of each professional group on the decision.

Professional Group	neutral 1	2	3	moderate 4	5	6	largest 7
Physicians							
Nursing staff							
RTs							

SLPs							

10 Matrix Change of tracheal cannula
Who is conducting the change of a tracheal cannula?
Please specify whcih professional group is involved and how often.

Professional group	Never 1	rarely 2	sometimes 3	often 4	always 5
Physicians					
Nursing staff					
RTs					
SLPs					

11 Communication management
Are you using the ACV (above cuff vocalisation) method?
If no, please jump tot he next question.

- o Yes
- o No

12 Matrix ACV
Who recommends the use of ACV?
Please rate the influence of each professional group on the decision.

Professional Group	neutral 1	2	3	moderate 4	5	6	largest 7
Physicians							
Nursing staff							
RTs							
SLPs							

13 Leak speech
Are you using leak speech for communication?

- o Yes
- o No

14 Matrix Leakage
Who recommends the patient to be decuffed?
Please rate the influence of each professional group on the decision.

Professional Group	neutral 1	2	3	moderate 4	5	6	largest 7
Physicians							
Nursing staff							
RTs							
SLPs							

15 PMV
Do you work with a speech valve that is competible with the MV, e.g. Passy Muir Valve or Primedi Phon Vent?
If no, please jump to the next question.

- o Ja
- o Nein

16 Matrix PMV
Who recommends the use of a PMV / Primedi Phon Vent?
Please rate the influence of each professional group on the decision.

Professional Group	neutral 1	2	3	moderate 4	5	6	largest 7
Physicians							
Nursing staff							
RTs							
SLPs							

17 Matrix Use of a one-way-speaking valve
Who recommends the use of a one-way-speaking-valve during spontaneous breathing (disconnected from the ventilator)?
Please rate the influence of each professional group on the decision.

Professional Group	neutral 1	2	3	moderate 4	5	6	largest 7
Physicians							
Nursing staff							
RTs							
SLPs							

18 Adjustment MV
Are you allowed to disconnect the patient from the ventilator?

- o Yes, alone
- o Yes, under supervision
- o No

Are you authorized to adjust the ventilator settings to use a PMV for instance?

- o Yes, alone
- o Yes, under supervision
- o No

19 Dysphagia management
Who is authorized to conduct swallow assessments with trach patients?
Multiple choice answers

- o Nursing staff
- o RTs
- o SLPs

Are you using the Modified Blue Dye Test (MBDT) for swallow assessments with trach paitents?

- o Yes
- o No

20 Secretion management
Who conducts Cough training measures (e.g. PEP-Valves, Cough Assist, manual techniques)?

Professional group	Never 1	rarely 2	sometimes 3	often 4	always 5
Physicians					
Nursing staff					
RTs					
SLPs					

21 Matrix Cough training
Who conducts secretion management (inhalations, use of secretion reducing medicines)?

Professional group	Never 1	rarely 2	sometimes 3	often 4	always 5
Physicians					
Nursing staff					
RTs					
SLPs					

22 Matrix Reduction of Secretions
Who recommends the use of pharmacologicel secretion reduction measures?
Please rate the influence of each professional group on the decision.

Professional Group	neutral 1	2	3	moderate 4	5	6	largest 7
Physicians							
Nursing staff							
RTs							
SLPs							

23 Matrix Medicines
Which active pharmaceutical secretion reducing ingredients are being used and how often?

Medicine	never 1	rarely 2	sometimes 3	often 4	always 5
Atropin					
Scopolamin					
Glyxopyrroniumbromid					
Botolinumtoxin					
Others					

24 Strategic Concepts
Are there written concepts (clinical pathways/SOPs/procedural instructions) for..?
Multiple Choice Answers
- o Weaning
- o Trach management
- o Dysphagia management
- o Decannulation
- o Communication management
- o Further:

25 Matrix SOP
Who is involved in the development of the above mentioned concepts?
Please rate the influence of each professional group on the decision.

Professional Group	neutral 1	2	3	moderate 4	5	6	largest 7
Physicians							
Nursing staff							
RTs							
SLPs							

26 FEES
The use of FEES (flexible endoscopic evaluation of swallowing) is
particularly important in the field of weaning, tracheostomy tube management and dysphagia management, and is regarded as an indicator of quality.
In this set of questions, the term FEES encompasses both LARYNGOSCOPY and ENDOSCOPIC SWALLOWING EXAMINATION.
Inspection of the trachea is listed separately as TRACHEOSCOPY, even though
both examinations are frequently performed together.

27 Use of FEES
Do you conduct FEES for every patient by default?
- o Yes, for every patient
- o No, not for every patient

28 FEES why
To what purpose do you conduct FEES?
Multiple choice answers
- o Inspection pharynx/larynx
- o Assessment Secretion management
- o Assessment Above Cuff Vocalisation/Use of a speaking valve /leakage ventilation
- o Assessement of swallowing different consistencies
- o Before oralisation
- o Before diet advancement
- o Assessement as a prerequisite for decannulation
- o Further:

29 Other diagnostic measures
Are there any other diagnostic methods that are being employed on the ENNR unit?

Multiple choice answers

- o Videofluoroscopy
- o Modified Barium swallow
- o Auscultation
- o HR-Manometry
- o Others:
- o No

30 Matrix FEES
Who is conducting the FEES?
Execution through

Professional group	Never 1	rarely 2	sometimes 3	often 4	always 5
Physicians					
Nursing staff					
RTs					
SLPs					

31 Tracheoscopy
Do you conduct a tracheoscopy for every patient by default?

- o Yes, for every patient
- o No, not for every patient

32 Tracheoscopy purpose
To what purpose do you conduct FEES?
Multiple choice answers

- o Inspection trachea/bronchii
- o Assessment Secretion management
- o Before Oralisation
- o Assessment trachea/trachestoma while changing the cannula
- o Retrograde assessment of subglottic structures
- o Assessement as a prerequisite for decannulation
- o Control of position of tracheal cannula (especially with fenestrated tubes)
- o Further:

33 Matrix Tracheoscopy
Who is conducting the tracheoscopy?
Execution through

Professional group	Never 1	rarely 2	sometimes 3	often 4	always 5
Physicians					
Nursing staff					
RTs					
SLPs					

34 Scores
Which of these scores are you using in trach and dyphagia management?
Multiple choice answers

- o Airway Care Score (ACS)

- Murray Secretion Score
- Penetration-Aspiration-Scale Rosenbek (PAS)
- Yale Pharyngeal Residue Scale (YPRS)
- Functional Oral Intake Score (FOIS)
- Bogenhausener Dysphagie Score (BODS)/Munich Swallowing Score (MUCSS)
- Andere:

35 Standard Page
Are there any other aspects or methods beyond these questions that have not been addressed, or any comments or observations regarding the work of speech-language pathologists or respiratory therapists in an ENNR weaning unit that you would like
to see included? I look forward to your comments and suggestions

36 Request for an Interview
Thank you very much for your participation. I have one more request:
If you work in a clinic or ENNR unit for weaning from mechanical ventilation where both professional groups—speech-language pathologists and respiratory therapists—are present, would you be willing to participate in a face-to-face interview?
The interviews are intended to explore the aspects and methods discussed here in greater depth. Particularly, I would like to learn more about your experiences with interdisciplinary collaboration between respiratory and speech therapists.
If you would be willing to do so, please feel free to contact me via email at katrin.eibl@uni-bielefeld.de
A short message, e.g., "Yes, I am available for an interview," is sufficient.
I would be very happy to hear from you. The interviews can take place via Zoom or Teams, or by phone if you prefer. You can find more information about the interviews at these links:
https://ww2.unipark.de/uc/Interview_Info/
https://uni-bielefeld.sciebo.de/s/97yVbdA1wZKUbLO
I look forward to hearing from you at:
katrin.eibl@uni-bielefeld.de

37 Last page
Thank you very much for you participation!
katrin.eibl@uni-bielefeld.de
